# Solidarity in disaster scholarship

**DOI:** 10.1111/disa.12657

**Published:** 2024-09-17

**Authors:** Ksenia Chmutina, Jason von Meding, Darien Alexander Williams, Jacob Remes, Wesley Cheek, Kaira Zoe Alburo‐Cañete

**Affiliations:** ^1^ School of Architecture, Building and Civil Engineering Loughborough University United Kingdom; ^2^ Florida Institute for Built Environment Resilience, M.E. Rinker, Sr. School of Construction Management University of Florida United States; ^3^ Macro Practice, School of Social Work Boston University United States; ^4^ Gallatin School of Individualized Study New York University United States; ^5^ Massachusetts Maritime Academy United States; ^6^ International Institute of Social Studies Erasmus University Rotterdam The Netherlands

**Keywords:** disaster scholarship, justice, knowledge production, solidarity

## Abstract

Disaster scholarship purportedly promotes disaster risk reduction and resists disaster risk creation, thereby deeply engaging with transboundary existential risks, justice, and political power. It is thus a commitment to humanity, and for it to become truly equitable and just, solidarity must lie at its heart. In this paper we connect solidarity with knowledge production and assess the implications of disaster scholarship and the relationships on which it is built. We offer a critique of the kind of research produced by neoliberal academic institutions and provocations for resistance through solidarity. We call on disaster scholars to use these prompts to reflect on their practice, research ethics, and their commitment to other human beings, inside and outside of the academy. Solidarity can help scholars to avoid the saviourism, self‐congratulation, and paternalism that are common in academia. Solidarity in disaster scholarship is a worthy endeavour precisely because it yields a concrete alternative vision of resisting disaster risk creation through knowledge production.

## 1 INTRODUCTION

*[I]n the long run the practice of solidarity proves much more advantageous to the species than the development of individuals endowed with predatory inclinations* (Kropotkin, 1902, pp. 17–18).

Disasters reflect global power structures. Consequently, we believe that the aim of disaster scholarship should be not only to contribute to disaster risk reduction, but also, more importantly, to help resist disaster risk creation. This position is generally accepted in disaster scholarship. Scholars argue that to resist disaster risk creation, it is critical to consider seriously transboundary existential risks, justice, and political power, as well as the role and knowledges of citizens and non‐state actors (Wisner, 2019; Chmutina, Cheek, and von Meding, 2021). The idea that we need to move beyond the universal concept of disasters grounded in Western ontologies (Gaillard, 2022) and accept that everyone's reality counts is gaining traction. But how do we build these shared considerations and move beyond theoretical debate? How do we make sure that disaster scholarship becomes a praxis between technical problem‐solving and commitment to humanity? Our scholarship will always be fraught with tensions, dilemmas, and, given the context of neoliberal academia, power struggles (Chmutina and von Meding, 2022); however, it can be made more equitable and just if solidarity is at the heart of our work.

Our operating setting presents some serious challenges. The structures and ideologies of the neoliberal academy produce isolation and competition and lead to the centralisation of university life, making it more difficult to build solidarity either within or without the quad (Crowther and Shaw, 2017). Disaster scholars (like those in many other disciplines) are often located within these very institutions, making it ever more urgent that we act in solidarity with each other before engaging with exploited and marginalised groups outside of the academy. Of course, there have been multiple efforts to confront the dominant paradigm of the neoliberal academy, and various examples of good practice exist—but by and large, our work happens within and is influenced by the conditions posed by neoliberal academia. Only a scholarly community with a depth of mutual care and commitment to one another will be ready to contemplate shared futures with the oppressed of the Earth. By thinking about solidarity, knowledge production, and the implications of our scholarship and the relationships on which it is built, this paper seeks to provoke questions, embrace discomfort, and promote other ways of contemplating solidarity as a way of moving disaster scholarship forward.

## 2 WHAT IS SOLIDARITY?

Solidarity is an important yet essentially contestable term. Over the years, it has been claimed by interests across the political spectrum, describing particular aspects of social and political interaction as well as prescribing their potential quality (Gaztambide‐Fernández, 2012). It is used both as a description of the actual level of common ground between individuals, and as a value that establishes normative connotations of mutual obligation and aid. Some argue that owing to such ambiguities, ‘solidarity’ has become a ‘sliding signifier [that] has no essential meaning and, like a glass, can be filled with multiple things’ (Apple, 2008, p. 244). Emerging from the nineteenth century social movements, in which solidarity signified counterhegemonic politics, and made prominent by Émile Durkheim (1933) in *The Division of Labour in Society* (a doctoral dissertation originally published in French in 1893), the term has increasingly assumed an ideological value, and its prominence in political theory and its relevance in many areas of social life are undoubtable. Its invocations, though, do not only represent the ideological values of the political left; they also ‘can [.. .] be found among conservative trade unions, within Catholic social teaching, and in nationalist and racist politics’ (Kip, 2016, p. 391).

Stjernø (2005) sees solidarity as a key concept that warrants reconsideration in an age of individualism. Scholz (2008) views ‘political solidarity’ as a commitment to social justice and as an effective response to injustice, oppression, or social vulnerability, highlighting it as a model of political and social participation. Bayertz (1999, p. 26) emphasises solidarity as ‘indispensable for a philosophy of morality and politics’. Brunkhorst (2005) asserts that it is a solution to social exclusion. Rorty (1989, p. xvi) underlines that solidarity is not what every person has in advance, it is ‘not discovered, but created via reflection’. Anarchists' conceptions of solidarity are also important to note: Michael Bakunin, Murray Bookchin, Noam Chomsky, and Peter Kropotkin all provide fresh, instructive perspectives on solidarity—see Nightingale (2015) for an in‐depth discussion of these four prominent thinkers. The contributions of the Black radical tradition are significant as well: Angela Davis, W.E.B. Du Bois, C.L.R. James, Claudia Jones, Cedric Robinson, and Walter Rodney, to name a few, help with understanding how to organise and theorise collectively (Du Bois, 2007; James, 2012; Lynn, 2017; Burden‐Stelly and Dean, 2022). Similarly, feminist interpretations of solidarity, including those by Sara Ahmed, bell hooks, Audre Lorde, and Chandra Mohanty, cannot be ignored. Abolitionists, both historical and contemporary, have expanded conceptions of shared fate by articulating visions of solidarity that extend to people who have been marked as nonhuman (Drescher, 2009; Kaba, 2021; Purdum et al., 2021).

Solidarity is not pre‐given and natural; it is socially constructed, affectively charged, and institutionally embedded. Morgan and Pulignano (2020) point to three sets of resources that are employed to explain and enact solidarity: (i) the language of morality deriving from a broader social vision about what ‘we’ share with others, and by contrast who may differ from ‘us’ (that is, ‘moral solidarity’ based on class, religion, and/or ethnicity); (ii) the use of the language and practice of political alliances and the formation of collective solidarities as necessary to achieve goals that can be widely shared on a pragmatic basis (that is, ‘political solidarity’ based on political calculations about the need for solidarity); and (iii) various rituals, symbols, and rhetorical appeals and vocabularies, such as marches, banners, and picket lines (that is, performative solidarity, in which effervescence is critical). These authors emphasise that ‘[i]t is important therefore to recognise that the language of solidarity is both a plea for recognition of the need for collective action but also an effort to shape this recognition in particular ways that conceals some key assumptions’ (Morgan and Pulignano, 2020, p. 23).

In contemporary labour movements, especially in North America, solidarity is based on an understanding of shared struggle. That is, to be productive solidarity, it must be an action that comes from a shared identity, the belief that our fights are different fronts of the same struggle (Lynd, 1984; Remes, 2012). Crucially, however, that shared identity is not primordial or based on immutable characteristics. Rather, it is built through experiencing the same conditions and participating in the same political struggles. In this conception, struggle both relies on and produces solidarity. In the context of disasters, this shared struggle may be that of survival in a disrupted society; disaster researchers have long noted the blossoming of post‐disaster altruism—see Erikson (1976, pp. 200–201) for an overview—which some have glossed as a sort of solidarity (Remes, 2016b). Yet, for post‐disaster altruism to blossom into longer lasting solidarity, it requires political work (McAlevey, 2016). For researchers to develop solidarity with those who bear the most disaster risk, we must embrace the shift from external ‘helper’ to an investment in shared dreaming and resistance.

This formulation of solidarity, as produced in and through shared struggles and affinities along political lines (as well as other forms of shared identities), creates potential for exploring new practices and articulating new arts of producing knowledge. Indeed, expressions of solidarity exemplified, for instance, in social movements are crucial sites for the construction of (other) knowledges, communities, and identities in relation to hegemonic forms of thinking and practice. Overall, solidarity is not about providing hope and charity to the impoverished and oppressed, but about supporting projects for social change, which inevitably are fraught with complexity (Power and Charlip, 2009).

### 2.1 Solidarity in academia and scholarship

Solidarity at work has long been considered essential to empowering labour in capitalist societies, enabling employees to build power in workplaces and sectoral and industrial bargaining, and shaping regulation of the labour market. There has been a noticeable decline in labour solidarity at work since the 1970s owing to the neoliberal changes of marketisation and privatisation, accompanied by legislation that undermines the ability of workers to engage in collective action (Richards, 2010; Morgan and Pulignano, 2020).

Solidarity among academic labour has been researched (see, for example, Moroz and Swabovski, 2017; Bieliauskaitė, 2021), and the recent waves of academic strikes (notably in the United Kingdom and the United States) make a conversation about solidarity in scholarship particularly apt, given that academic work has been made routine, more precarious, and more casualised (Herbert, Apkarian, and van der Naald, 2023). At its best, the academic labour movement can be understood as a fight against a version of academe that prizes individualised intellectual work, discourages solidarity, and encourages belonging to little groups that hoard opportunities, such as research funding (see, for example, Nelson, 1996; Burke, 2023; Koruth, 2023). Furthermore, neoliberal academia demands scholars to be ‘impartial’ sources of isolated knowledge who do not utilise their political views and moral values in teaching their students (Genao, 2021). This institutional arrangement precludes the inventive, transdisciplinary research questions and methodologies required to solve pressing local and global challenges (McKittrick, 2021). Scholarship is thus under attack by a new kind of irrationalism often fostered and funded by regressive sections of the ruling class, while creative activities and ideas are subordinated to forms of information management (Wark, 2021).

The contemporary university is frequently seen as being driven by an individualising neoliberal ethic (Collini, 2012; Loveday, 2018), promoted as a virtue while encouraging ‘toxic individualism’ (Gill, 2018, p. 106). What is more, critiques have long been made of academia and universities as spaces of whiteness (Bhopal, 2016; Arday and Mirza, 2018; García Peña, 2022), patriarchy (Marshall and Witz, 2004), able‐bodiedness (Burke and Byrne, 2020; Sheppard, 2021), and middle‐classness (Reay, Crozier, and Clayton, 2009). Those who ‘perish’ rather than ‘publish’ are labelled as individuals who lack resilience or grit. Burton (2021, p. 24) summarises this well in a paper titled ‘Solidarity, now!’: ‘The omnipresent atmosphere of competition, judgement, fear, anxiety, and risk combines with the encouragement to boast and emboldens the idea that ego and selfishness are vital career attitudes, resulting in an academy which is deeply and acutely unkind, unforgiving, and uncaring’. Yet, there is a lack of continuous methods of opposition to these conditions. Burawoy (1979, pp. 11–12), researching the factory floor, states that ‘the normative relationship between reward and effort, and the underlying conflict, is taken as given’. This is true in academia as well. When confronting the current bureaucratic, neoliberal university, it is important to remember that the process of recruiting, training, and promoting the technocratic elite creates its own kind of solidarity, although maybe not the one we are seeking (Öniş, 1991).

So, is it possible to counter—or even resist—the violence of neoliberal academia through solidarity? Can this recognition of shared humanity and personhood, and the acknowledgement that we exist and feel both as collectives and autonomous social actors, free us to establish more meaningful relationships with each other, the risk‐bearing communities, and our environments that we purport to serve? Solidarity through cooperation can help to push back against an ideology that seeks separation, isolation, and alienation; it would also promote the idea that intellectual endeavours are a team game and not an individual competitive sport. Such cooperation would require an open dialogue based not on hierarchical power, but on respect and trust. This would eventually lead to shared values grounded in kindness and care that could then be mobilised to enable practical and conceptual cultural shifts in academia to change the way we work and engage with one another and with research partners and participants. This dialogue does not have to lead to a consensus: conflict in conversation can be significant in the process of recognising difference in meaningful ways (Hill, 2019); and this is important for solidarity.

Solidarity can also provide comfort (Alburo‐Cañete et al., 2022), allowing for the navigation of the ethical‐political in ways which are open to multiple possibilities that are frequently important in scholarship (instead of choosing a ‘right’ or ‘wrong’ answer). It permits a move away from conventional or dominant academic attitudes towards such issues as the appropriate degree of distance between movement and researcher in alternative, participatory approaches (such as queer public sociology, participatory action research, activist research, and politically‐engaged ethnography) that purposively eschew notions of ‘impartiality’ or ‘objectivity’ and ask scholars to think reflexively about their research practices (Brem‐Wilson, 2014). We might work ‘with love and rage’, defying a market‐driven academy to assert our ethical and political agency (James, 2013, p. 211).

What does it mean to do scholarly work in solidarity with other scholars as well as with those at risk of disaster? The first step is to approach knowledge creation as a shared endeavour and to move away from treating subjects as mere sources of data. Rather, we must learn directly from those ‘closest to the trouble’, by taking seriously the knowledge created by them and their learning (Remes and Horowitz, 2021, p. 3).^1^ Remembering the inherent political valence of ‘solidarity’ and the way the term has developed its meaning within social movements can help scholars. Conversely, however, we must also recognise that experiential knowledge and its translation into scholarship will always necessarily be incomplete. Scholarship on and experiential knowledge of disaster each contribute different things, and so scholarship in solidarity recognises the potentially generative incompleteness of translation (Remes, 2016a; Watanabe, 2021).

## 3 WHAT DOES SOLIDARITY MEAN FOR DISASTER SCHOLARSHIP?

Solidarity often occurs within groups that have something in common. ‘Community’ is perhaps one of the most researched topics in disaster scholarship, ranging from communities' resilience and participation to their exposure and vulnerability. The idea of community is sprawled across documents of best practices and penetrates international, national, and local policies. The word ‘community’ is used nine times in the 25 text pages of the *Sendai Framework for Disaster Risk Reduction 2015–2030* (UNISDR, 2015, pp. 3–27). Yet, ‘community’ is rarely unpacked as one of the values closely connected to anti‐capitalism (Wright, 2021); consequently, solidarity, which in the context of anti‐capitalism is closely linked to community, is not discussed either (Cheek, 2020).

Hall (1990, p. 222) described community as the overlapping, intermingled, and consistent tangle of mutually understood ‘common historical experiences and shared cultural codes’. This tangle was broad and interconnected, so while it could change over time, it was a relatively stable formation. For Hall (1990), it was possible for there to be differences, even contradictions, within a community; however, the underlying unity preserved the arrangement. Freire (1998) understood communities as being nested within each other, fractal and reducible, but recognisable to those who were members. Outsiders would be tempted to simplify the boundaries of a community, to reduce them both in complexity and intricacy. For Freire (1998), community was in a constant tension; being defined and delimited by outsiders, while being negotiated and comprehended by insiders. Gramsci (1971) too was cautious about the idea of community, showing that it could often be used by outsiders as a tool of legitimisation. The community was frequently asked simply to acquiesce to the demands of power and thereby be recognised as ‘a community’.

Disaster scholars have struggled with this notion of community. Aldrich and Meyer (2015, p. 255) have defaulted to the community as a ‘geographically defined area’. This is understandable as legally, politically, and practically, geographic definitions hold a great deal of weight. Cheek (2020) revealed an ambivalence on the part of those grouped together as a community about understanding themselves in those terms. Perhaps Simone (2004) provides a path forward in linking these ideas of community and solidarity. In his conception the community is something whose borders, history, and intricacies are mutually understood by those within it (Simone, 2004, p. 410).

Solidarity suggests the idea of collective power—‘united we stand, divided we fall’—grounded in the concepts of unity and cooperation that are motivated ‘not exclusively by an instrumental concern with narrow individual self‐interest, but by a combination of moral obligation to and concern for others’ (Wright, 2021, p. 29). These attributes are often exposed in a disaster: people in affected areas frequently come to each other's aid in striking and unselfish ways (see, for example, Remes, 2016b; The Care Collective, 2020; Faas, 2022); a strong sense of community also serves to make people less vulnerable (Aldrich, 2012; Aldrich and Meyer, 2015; Klinenberg, 2015). Research has suggested that the US labour movement built power and practices of solidarity that contributed not just to better health outcomes and behaviours during the height of the COVID‐19 (coronavirus disease 2019) pandemic (Dean, Venkataramani, and Kimmel, 2020; Dean et al., 2021, 2022; Vachon, Wallace, and Li, 2023). Of course, the sense of community and solidarity can manifest itself as inegalitarianism and exclusionism (that is, insiders versus outsiders); for instance, nationalism often functions in this way (Hechter, 2010). Thus, ‘while the value of community does figure in emancipatory ideals, much depends on precisely how it is articulated vis‐à‐vis the values of equality and democracy’ (Wright, 2021, p. 30).

Mutual aid, one of the key steps towards solidarity, is a powerful force. It exposes the failures of the current system and reveals alternatives: its long tradition of pulling together resources (Kropotkin, 1902) has, over the decades, ranged from addressing Black exclusion from white infrastructures to creating Black alternatives to urban planning and public education efforts, and from supporting workers on strike to breaking stigma, shame, and isolation (Wilder, 1998; Spade, 2020; Williams, 2023). Being able to get help in a crisis is often a condition for being politically active because it is very difficult to organise when also struggling to survive (Spade, 2020). What is more, it is through mutual aid that we start working together and learning about experiences different from our own, thereby building solidarity across those differences.

### 3.1 Finding the ‘we’ in disaster scholarship

What is often missing in academia is this sense of community—a collective identity, purpose, and vision (as our performance, while based on the rhetoric of collaboration, evaluated using individual metrics and indices such as citations and grant income); yet, it is this ‘we’ that permits us to reclaim our interdependence. It allows for the sense of something that is made by *us* (as opposed to, say, a god or a political leader) (Nancy, 2016). Such positioning of ‘we’ also calls for a powerful question of who ‘we’ are today, what constitutes ‘we’—that is, the ontological questions of the political (Critchley, 2009)—and how ‘we’ could and should resist disaster risk creation.

One way to form a ‘we’ may be to recognise that disaster researchers share hardships with others. Our emphasis above on the alienating aspects of neoliberal academia shows that scholars too are victims of capitalism, racism, misogyny, and other structures of oppression. Nor are academics invulnerable to hazards. Indeed, some scholars find their way to disaster research because they have personal experience as disaster sufferers (English, 2015; Chmutina et al., 2024). In scholarly traditions that prize pretences of objectivity, it can feel radical to admit that disaster researchers are also victims or potential victims of disaster—that is, that we are also people living on Earth—but to start with our shared humanity is the starting point for solidarity. Nowhere is the necessity for our interconnection clearer than in consideration of how we will collectively navigate the impacts of climate change (Mann and Wainwright, 2020; Ferdinand, 2022).

However, it is also important to be mindful of our presumptions regarding who the ‘we’ are in collectivist discourses of solidarity. As raised by postcolonial feminist scholar Chandra Mohanty (2003), differences and commonalities exist in relation to and in tension with each other, even in spaces where notions of sharedness are pronounced. Black feminist scholarship, for instance, has refuted understandings of solidarity as ideological uniformity. In the context of anti‐racist movements in the US, Black solidarity has frequently taken on patriarchal and homophobic forms. Black feminists have therefore committed to community work and organising against racism as a form of solidarity among Black communities, but at the same time, problematised sexism and heteronormativity have permeated these spaces. Collins (2017) refers to these acts as *flexible solidarity*, a concept that advances non‐essentialist/non‐homogenising and more fluid understandings of solidarity that help enable the transversal politics necessary for coalition‐building.

Reflexivity and mindfulness of positionality and situatedness of knowledge remain crucial elements for forging solidarities across divisions of race, class, gender, beliefs, and so on. Such transdisciplinary, transnational, transboundary forms of solidarity are rooted in place and history yet are intimately connected to envisioning alternative ways of being with and among broader communities (Harcourt and Nelson, 2015).

Solidarity can also open an inquiry into the terrain of human collectivity, which should be a foundation for disaster scholarship—that is, recognition of interdependencies and vulnerabilities (De Lissovoy and Brown, 2013; von Meding and Chmutina, 2023; Wisner, 2025). The problem is that instead, disaster scholarship is notorious for seeing people affected by disasters as ‘subjects’ of research, thus creating a division between ‘us’ and ‘them’. This leads not only to a myriad of ethical issues, but relationally it creates isolation, resulting in a retreat into a divisive, competitive, privatised creation of knowledge—the kind that capitalism loves (Brookfield, 2004). Much of disaster scholarship fails to engage in a reflexivity necessary for researching the ‘other’ (Klutz, Walker, and Walter, 2020), and instead seeks ‘pain stories’ (Tuck and Yang, 2014, p. 29) from marginalised communities that, while often arising from good intentions, further marginalise and position people as ‘singularly defective and powerfulness to make change’. This is evident, for example, in the way that vulnerability is framed as weakness—and such research invariably ‘others’, even when well‐intentioned.

This troubling of the ‘we’ extends to relationships between colleagues based in the Global South (that is often the focus of disaster scholarship), whose capacity many Global North academics are striving to ‘develop’ without seeing these actions as an extension of neo‐colonialist and imperialist practices. This has been challenged by many disaster scholars—see the *Disaster Studies Manifesto* ^2^ as an example—yet more action is required if we are to achieve more respectful, reciprocal, and genuine relationships among researchers.

Often, the idea of solidarity relates to the idea of hardship, as opposed to the idea of safety and surplus that feed body and soul and encourage private demands. But if, following Tuck and Yang (2014), we seek to move beyond ‘pain stories’, it may be our task to extend solidarity beyond survival and scarcity and the disaster research agenda beyond merely the hardship, shared or not. Disaster scholars focusing, for instance, on the vulnerability of women in flood‐prone areas, hopefully realise that racism, colonialism, ableism, police violence, the foster care and health care systems, and more are all causes. Yet, the research hardly ever goes beyond ‘helping women’ in an atomised material sense, instead of furthering the help in terms of creating and encouraging useful pathways for resistance. Moreover, the solutions are often one‐dimensional, driven by disciplinary silos within which many scholars operate. Furthermore, even though a scholar frequently experiences a dissatisfaction with injustice, the realisation of it does not follow as it contradicts the ‘rules’ of academia (that is, to remain objective and impartial). The ‘solutions’ proposed by academic researchers are then translated into on‐the‐ground interventions that often seek to ‘empower’ those at risk but do not challenge the power relations that have produced risk in the first place (Clark‐Ginsberg, 2021).

Many agree that the causes of disaster are rooted in oppression. This is important in the context of solidarity because it is built on sharing knowledge and communication to strengthen struggles against capitalism, patriarchy, racism, and colonialism. However, the more difficult the experience of oppression, the harder it is to share, meaning that solidarity is often most scarce precisely when it is most needed (de Sousa Santos, 2018). There is a lot of diversity in the experiences of oppression—and the oppressors are consistent in ensuring that this diversity turns into isolation. This is exactly why such isolation needs to be superseded by reciprocity, cooperation, and solidarity based on and in differences.

### 3.2 Steps towards solidarity

In this paper we are urging disaster scholars to reflect on their practice, on their research ethics, and on their commitment to other human beings, inside and outside of the academy. If you are reading this and want to take action, we want to share some ideas as to what you can do. In recommending steps towards solidarity, we recognise that within the neoliberal university, time is in short supply for this often gruelling, counter‐institutional work. These acts are necessarily disruptive, and we know the risks of disrupting norms—but it gives us all the more reason to foster solidarities. Part of our political work is therefore to find entry points to the creation of enabling environments. Co‐reflecting on our various experiences and practices in disaster research, we have identified the following insights that could contribute to working in and through solidarity; these are not exhaustive, but they can be considered as starting points for advancing the kind of liberating scholarship we envision:

- *Commitment to engaging multiple systems of knowledge*. Here, we are talking about theoretical openness and a shared understanding of why people do not have what they need. Too often, disaster scholarship focuses on disasters as technical problems that require technocratic solutions—for a critique, see Chmutina et al. (2023); this is true not only of disaster scholarship produced by many natural scientists and engineers, but also of scholarship by many social scientists who insist on the power of quantification and generalisation (Chmutina and von Meding, 2022). This inevitably results in apolitical, ‘objective’ scholarship that does not conceptualise how and why it influences and is influenced by much broader sociopolitical and economic contexts. Such scholarship can easily be co‐opted into capitalist narratives of efficiency and economic well‐being, with funders actively encouraging projects that do not fight for justice, but instead invent (and patent) new ways of managing marginalised people and social problems. This kind of scholarship serves to gloss over the social and political conditions producing crises. Shared understanding can help scholars to remain oppositional to the status quo and cultivate resistance, rather than becoming complementary to the economic forces that create disasters in the first place.
- *Scholarship for liberation, not charity*. To address root causes, disaster scholarship needs to focus on efforts that both support survival efforts (that is, reduce and protect from disaster risk) and collude in powerful forms of resistance to the systems that create risk initially. Too often, scholarship only concentrates on the former, while mainstream understanding of how to support people in crisis relies on frameworks of charity, relief, and aid. These terms usually refer to those in power making decisions about who gets the help, what the limits are to it, and what strings are attached. This kind of help is not designed to get to the root causes of poverty and violence (Foley, 2010; Seymour, 2012)—and academics are often complicit in supporting this by putting band‐aid solutions on massive wounds created by the greed of the powerful. Furthermore, scholarship that concentrates on reinforcing charity promotes hierarchies of knowledge and frequently, although unintentionally, also promotes hierarchies of wealth and leaves vulnerability to be framed as a label of weakness (and sometimes laziness and immorality).
- *Solving problems through collective research and action*. Working together develops skills for collaboration, participation, and decision‐making. This may sound like an obvious step as collaboration is often positioned as a virtue of scholarship—yet, here we are talking about meaningful collective research and action, which allow for working across differences, addressing conflicts, giving and receiving feedback, and appreciating that it is not just the most senior academic in the group that can do this. This entails conducting research and sharing authorship with non‐academics, and with those whose positionality frees them from some of the aforementioned perverse academic incentive structures (Guidry, 2017; Purdum et al., 2021; Miles, Renedo, and Marston, 2022). This moves us away from expertise‐based scholarship to anti‐authoritarian scholarship, demonstrating how we can do things together in ways that we were told not to imagine, without coercion. Such problem‐solving requires unlearning conditioning that academia is full of and generating boldness—but it will also lead to learning new skills and capacities.
- *Listening as a political act*. This proposition may seem strange, but as Taylor (2021, p. 101) notes, ‘to listen is to act; of that, there's no doubt. It takes effort and doesn't happen by default. As anyone who has been in a heated argument—or who's simply tried to coexist with family members, colleagues, friends, and neighbours—well knows, it's often easier not to listen. We can tune out and let others’ words wash over us, hearing only what we want to hear, or we can pantomime the act of listening, nodding along while waiting for our turn to speak. Even when we want to be rapt, our attention wanes. Deciding to listen to someone is a meaningful gesture. It accords them a special kind of recognition and respect’. How we hear someone relates to their gender, race, sexual orientation, age, physical ability, and wealth; in other words, our hearing relates to and often reinforces social prejudices. By listening in this manner, we can work to avoid what Morimoto (2023, p. 5) has referred to as ‘an unintentional extractivist’: a researcher who passively hears, but only to use that information for their own promotion. We thus need to learn how to strain our hearing to listen to the voices that are drowned out by the powerful. An inclusive and vibrant public sphere in which we can all listen to one another is the foundation for collectivity and, consequently, solidarity.
- *Go beyond critique*. Critical perspectives in disaster scholarship have undoubtedly played a role in foregrounding crucial issues that have for so long been overlooked in the creation of risk: social inequalities, unjust systems, and power imbalances in the production of disaster scholarship, to name a few. However, embodying solidarity also means going beyond critiquing towards making visible alternatives and possibilities that are often erased in conventional scholarship. As we embark on processes of learning (and unlearning) within and through acts of solidarity, we need to recognise that the research that we do is a performative practice: it is an act of bringing realities into being. As articulated by Gibson Graham and Roelvink (2010, p. 342), as a form of a mandate, (disaster) scholars are called to ‘read the potentially positive futures barely visible in the present order of things’, amplify them, find ways to strengthen them, and move them along.
- *Engaged scholarship and accountable activism*. Many scholars do little beyond the accumulation and communication of evidence—yet some argue that civil disobedience is a reasonable and ethical activity for scientists to undertake (Capstick et al., 2022). Observation, discovery, analysis, and presentation are the central goals of both scholarship and activism—but it is the combination of scholarship and activism that allows the politics of recognition to flourish (Gilmore, 2022). This is important because solidarity requires organised actions, and organising inevitably is laden with differences, characters, purposes, and interests. This organised action needs to go beyond advocacy and involve acts of conscience that seek to disrupt and resist the status quo or to lobby changes in laws and practices. There are numerous historical examples of scientists and scholars taking the stakes of their work seriously via activism: W.E.B Du Bois' Panafricanism and peace activism; Angela Davis' revolutionary work with incarcerated Black and Puerto Rican women; Frantz Fanon's membership of the decolonial Algerian National Liberation Front; and Walter Rodney's founding of the Guyana's Working People's Alliance. We are witness to a growing number of scientists becoming involved in various protests (notably, many climate scientists are involved in the Extinction Rebellion protests in the UK). The impact of collective action within social movements is also noted by the Intergovernmental Panel on Climate Change, which has played a substantial role in pressuring governments to create new laws and policies (Shukla et al., 2022). All this being said, not every scholar has the same freedoms or level of professional/personal risk when it comes to resisting the status quo. Collective strategic action can help us to look out for one another and avoid doing harm ourselves.
- A *community built on friendship*. Often a community is represented by a leader and thus embodies them. However, to build solidarity, the community needs to work against rigid hierarchy: it needs to be ‘all united’ while eschewing centralised power. This is possible if the idea of community is built around friendship, because friendships are always built in opposition to dissolution of a singularity and are fuelled by disputes, concession, and sharing. McKittrick (2021, p. 73) reminds us that ‘[f]riendship is hard freedom. Maybe friendships effectuate consciousness and liberation and possibility’. Derrida (2020) writes about the temporality of friendship: the future in friendship is an experience of slow protraction, of distention of future tense; the past of friendship is the time of recollection, testimony, and narration, that is, experiences deeply linked with memory, or recalling time together or in solitude. Friendship is thus rooted in the passage of time, which allows friends to be accountable to each other. Mbembe (2021) also reiterates the importance of commitment to learning and remembering together, not as a matter of charity or compassion, but as a condition of survival of our world. Could solidarity be interpreted in a similar manner? Just like friendship, disaster scholarship is about the past and the future. It largely focuses on exposing the root causes of disasters (the past) and finding ways of addressing them (the future); rarely, though, do we acknowledge the shared experiences that stem from these root causes.
- *Building a community of care*. Scholarship often only recognises the final, outward‐looking evidence of production: a publication, a large funding grant, or an event. The caring labour that is always required to build meaningful collaboration is frequently ignored, even among the most politically aware scholars. We have not been taught to notice or care about how things went along the way, whether every individual's capacity for confronting the next challenge was improved, whether they were destroyed through burnout or damaging collaborative dynamics, or how researching disasters actually affects scholars as persons. We must therefore build stronger structures that allow for participatory, transparent decision‐making structures and cultures of care and principled action. A scholarly community of care is one in which everyone, from administrative support and students to early career researchers and scholars in more precarious positions, knows how to raise concerns, propose ideas, and put decisions into practice. It is one in which privilege is acknowledged and comes with responsibilities towards those bearing more risk. Consequently, we must avoid hierarchy, a lack of clarity, over‐promising and under‐delivering, elitism, and competition. Instead, we must recognise co‐leadership, humility, transparency, shared decisions about workloads, planning, generosity, cooperation, and accountability.
- *Comradeship not allyship*. For disaster scholarship to act in solidarity, it is important to understand and embrace the difference between allyship and comradeship (Dean, 2019; Klutz, Walker, and Walter, 2020; Southam, 2021). In recent years, calls for allyship, including those in academia, have been on the increase. Allies are generally privileged people who do not agree with systems of oppression. But too often being an ally is considered as an identity, which comes with a set of instructions (such as how‐to guides and a set of pointers). The ‘politics’ in these allyship how‐tos consists of interpersonal interactions, individuated feelings, and mediated affects, centring the ally's own individual disposition, attitude, and behaviour, rather than the struggle that remains opaque (Dean, 2019). Comradeship, meanwhile, ‘indexes a political relation, a set of expectations for action toward a common goal. It highlights the sameness of those on the same side—no matter their *differences*, comrades stand together’ (Dean, 2019, p. 13; authors' emphasis). Differences are important here because these are inevitable in academia, owing to our training, disciplinary, and personal background, as well as our positions. Comradeship is critical for solidarity because it is through comradeship that the vision for the shared future is created, although comrades may not like each other and even disagree. Comrades can thus count on each other because they are accountable to each other. ‘Where the ally is hierarchical, specific, and acquiescent, the comrade is egalitarian, generic, and utopian’ (Dean, 2019, p. 30)—and it is the latter that we need to cut through the determinations of the everyday and carry out scholarship that makes a difference and challenges the status quo.
- *Be of use*. Lastly, a core aspect of acting in solidarity with someone else is that they need and want your actions. For disaster scholars to act in solidarity with communities at risk of disaster means to generate scholarship that is useful to people in those communities. That does not mean producing work that will be uncontroversial or universally welcomed in these communities at risk. Sometimes the best way to act in solidarity is to be critical or challenging. But scholars seeking to work in solidarity should strive to create knowledge that is usable by the communities that need it.

Practising the steps outlined above can help disaster scholars to consider better our mutual relationship (among researchers and the researched) as well as the methodological and ethical principles that our scholarship spawns. It can also highlight that the type of knowledge that we produce is directly shaped by our motivations and ends, prompting recognition of the need for a researcher to ‘interact politically with a world whose realities of social exclusion and inequality demand a proactive role’ (Santos, 2012, p. 142). Moreover, solidarity among scholars can teach how to be deliberate with connections between disasters and social justice and to support better transformative learning while reconsidering the role of politics in the classroom (Genao, 2021; Chmutina and von Meding, 2022). Solidarity in disaster scholarship acknowledges that disasters are not an event but a sociopolitical process that is continued and consistently present.

## 4 CONCLUDING REMARKS

The idea of solidarity in disaster scholarship may indeed be decried as naive, because scepticism is often assumed to be the only ethical position worthy of intellectual discourse. As Berlant (2011, p. 122.) writes, ‘[e]ven Adorno, the great belittler of popular pleasures, can be aghast at the ease with which intellectuals shit on people who hold a dream. Dreams are seen as easy optimism, whilst failures seem complex’. But dreaming is a political act, an ‘integral part of the historico‐social manner of being a person … There is no change without dream as there is no dream without hope’ (Freire, 1998, p. 90). So, it is the dream of solidarity that should be driving us if we want disaster scholarship to be effective and relevant, given its complexity and its multifaceted nature spread across a multitude of disciplines. We doubt that our peers would disagree that, as disaster scholars, we are all broadly committed to addressing the rights, needs, and interests of those impacted by disasters. Many of us may disagree on how to achieve this, perhaps because of our ideological beliefs or disciplinary training. Regardless, this commitment requires asking diverse questions—many of which are rooted in discussions about power—and finding answers that are frequently uncertain and unwelcomed. Thus, in pursuing this goal, we owe each other an abiding respect that permits learning and subsequent moral progress towards solidarity.

Solidarity can help us to avoid the saviourism, self‐congratulation, and paternalism that are common in academia. This is particularly prominent in the disaster context, especially in the Global South, where many Western academics who ‘collaborate’ with local researchers use their presumed superiority (often because it is them who secured funding) to ‘remake’ these people and places. They would replace old, dysfunctional ways of being with ‘smarter’ ones, offering ‘innovations’ that, although often unintentional, nevertheless eliminate public goods, permanently displace vulnerable residents, and devastate social infrastructure (Servet and Tinel, 2020; Alderson, 2021; Stein, Cunningham, and Carmody, 2021).

Solidarity can also help us to navigate what Gilmore (2022, p. 448) calls a ‘consistent inconsistency conundrum’: ‘it is *consistently* true that the engaged scholar of whatever political conviction works in the unavoidable context of dynamics that force her into self‐conscious *inconsistency*; she must at times confirm and at times confront barriers, boundaries, and scales’. Plenty of bad research is produced despite the best intentions. Traversing this is difficult; when working in solidarity, however, it becomes clearer how and why some attempts to intervene in a particular historical–geographical moment of injustice may change what others do and think.

Solidarity is what builds and connects large‐scale movements. Too often, disaster scholarship is urged to be single discipline‐oriented, framing its message around ‘deserving’ people within the population on which it focuses, and using scholarly approaches palatable to academia and funding elites. This kind of compromised work is not done in solidarity with the oppressed. The people who are the real experts on the risk that they bear are left out—as are the most uncomfortable questions. Solidarity across issues and populations is what can make disaster scholarship relevant and powerful. We do not argue against developing specialist knowledge; instead, we favour the application of this knowledge in more inter‐ and trans‐disciplinary ways and with respect for other knowledges (be those scientific or non‐scientific) as well as grounding our knowledge in reality—and solidarity. Without such a linkage, we end up with disconnected disciplines (some of which are perceived to be ‘better’ than the other (see McKittrick (2021)), undermining one another, competing for attention and funding, not backing each other up, and not doing research that can help in resisting the systems that create disasters. Indeed, we set ourselves up potentially to harm the very communities that we say we want to support. Solidarity is an evolving commitment to justice that emerges from experiencing complex realities of injustice; understanding such experiences thus requires collaboration to ensure that our scholarship contributes to a broader vision of social transformation rather than furthering our careers and ‘objective’ knowledge.

We do have to be careful, though, in that we do not see solidarity as an end in itself. Solidarity always remains ‘an uneasy, reserved, and unsettled matter that neither reconciles present grievances nor forecloses future conflict’ (Tuck and Yang, 2012, p. 3)—and can be used to reinforce and reproduce paternalistic relationships, inequalities, and exploitation as well as bullying through the idea of being a ‘better’ scholar (Gagliano, 2021; Alburo‐Cañete et al., 2022). Hence, we need a kind of solidarity that can navigate the complexities of privilege rather than being one that reinforces ‘insiders’ and ‘outsiders’. We need to think once again how and by whom the research is done, and to recognise that, if solidarity is practiced as a way of assuaging white—or other forms of—guilt that stem from certain privileges (De Lissovoy and Brown, 2013), this is not solidarity, because good intentions are not enough to build relationships. Practising solidarity involves re‐evaluating interdependencies in ways that can be deeply uncomfortable or even contentious; disaster scholarship built on solidarity is a situated epistemology that asks scholars to think reflexively about our research practice, the roles we play, the interests we serve, and how to go about our roles with care.

Disaster scholarship built on solidarity is scholarship that can truly resist disaster risk creation and prevent harm. However, this must be done with those who are being harmed and with those who understand the context; solidarity can only be achieved if our scholarship is grounded in intergenerational, interdisciplinary, and intercultural dialogues, embodies a sense of legacy, and establishes as legitimate the expectation that academics, activists, community members, and many others can and do contribute to the production of knowledge. Solidarity in disaster scholarship is a worthy endeavour precisely because it brings forth an alternative vision of resisting disaster risk creation through knowledge production, revealing in the process what needs to bend and what needs to break in order to make room for scholarship that is not yet here. This is the kind of scholarly community that might make rebellion possible (García Peña, 2022). Remember that solidarity is an action—so every time we say ‘I am in solidarity with… ’, we also need to ask ourselves: what is our praxis?

*I was going to die, if not sooner then later, whether or not I had ever spoken myself. My silences had not protected me. Your silence will not protect you. But for every real word spoken, for every attempt I had ever made to speak those truths for which I am still seeking, I had made contact with other women while we examined the words to fit a world in which we all believed, bridging our differences. And it was the concern and caring of all those women which gave me strength and enabled me to scrutinize the essentials of my living* (Lorde, 2012, p. 41).

## ETHICS STATEMENT

This paper reports analysis of secondary sources.

## Data Availability

Data sharing not applicable to this article as no datasets were generated or analysed during the current study.

## ENDNOTES

^1^
See also *Power, Prestige & Forgotten Values: A Disaster Studies Manifesto* at https://www.radixonline.org/manifesto-accord (last accessed on 19 August 2024).

^2^

*Power, Prestige & Forgotten Values: A Disaster Studies Manifesto* calls for rethinking our research agendas, our methods, and our allocation of resources. For more information, see endnote 3.

## References

[disa12657-bib-0001] Alburo‐Cañete, K.Z. et al. (2022) ‘(Dis)comfort, judgement and solidarity: affective politics of academic publishing in development studies’. Third World Quarterly. 43(3). pp. 673–683.

[disa12657-bib-0002] Alderson, P. (2021) ‘Health, illness and neoliberalism: an example of critical realism as a research resource’. Journal of Critical Realism. 20(5). pp. 542–556.

[disa12657-bib-0003] Aldrich, D.P. (2012) Building Resilience: Social Capital in Post‐Disaster Recovery. University of Chicago Press, Chicago, IL.

[disa12657-bib-0004] Aldrich, D.P. and M.A. Meyer (2015) ‘Social capital and community resilience’. American Behavioral Scientist. 59(2). pp. 254–269.

[disa12657-bib-0005] Apple, M. (2008) ‘Can schooling contribute to a more just society?’. Education, Citizenship and Social Justice. 3(3). pp. 239–261.

[disa12657-bib-0006] Arday, J. and H.S. Mirza (2018) Dismantling Race in Higher Education: Racism, Whiteness and Decolonising the Academy. Palgrave, Basingstoke.

[disa12657-bib-0007] Bayertz, K. (1999) ‘Four uses of “solidarity”’. In K. Bayertz (ed.) Solidarity. Dordrecht, Kluwer. pp. 3–28.

[disa12657-bib-0008] Berlant, L. (2011) Cruel Optimism. Duke University Press, Durham, NC.

[disa12657-bib-0009] Bhopal, K. (2016) The Experiences of Black and Minority Ethnic Academics: A Comparative Study of the Unequal Academy. Routledge, London.

[disa12657-bib-0010] Bieliauskaitė, J. (2021) ‘Solidarity in academia and its relationship to academic integrity’. Journal of Academic Ethics. 19 (September). pp. 309–322. 34093099 10.1007/s10805-021-09420-6 PMC8165131

[disa12657-bib-0011] Brem‐Wilson, J. (2014) ‘From “here” to “there”: social movements, the academy and solidarity research’. Socialist Studies. 10(1). pp. 111–132.

[disa12657-bib-0012] Brookfield, S.D. (2004) The Power of Critical Theory: Liberating Adult Learning and Teaching. Jossey‐Bass, San Francisco, CA.

[disa12657-bib-0013] Brunkhorst, H. (2005) Solidarity: From Civic Friendship to a Global Legal Community. MIT Press, Cambridge, MA.

[disa12657-bib-0014] Burawoy, M. (1979) Manufacturing Consent: Changes in the Labor Process Under Monopoly Capitalism. University of Chicago Press, Chicago, IL.

[disa12657-bib-0015] Burden‐Stelly, C. and J. Dean (2022) Organize, Fight, Win: Black Communist Women's Political Writing. Verso Books, London.

[disa12657-bib-0016] Burke, C. and B. Byrne (2020) Social Research and Disability: Developing Inclusive Research Spaces for Disabled Researchers. Routledge, London.

[disa12657-bib-0017] Burke, L. (2023) ‘3 takeaways from the Rutgers strike’. Higher Ed Dive website. 28 April. https://www.highereddive.com/news/3-takeaways-from-the-rutgers-strike/648657/ (last accessed on 19 August 2024).

[disa12657-bib-0018] Burton, S. (2021) ‘Solidarity, now! Care, collegiality, and comprehending the power relations of “academic kindness” in the neoliberal academy’. Performance Paradigm. 16. pp. 20–39.

[disa12657-bib-0019] Capstick, S. et al. (2022) ‘Civil disobedience by scientists helps press for urgent climate action’. Nature Climate Change. 12 (September). pp. 773–777.

[disa12657-bib-0020] Cheek, W. (2020) ‘The paradox of community involvement: rebuilding Minamisanriku’. Disaster Prevention and Management. 29(6). pp. 893–907.

[disa12657-bib-0021] Chmutina, K. , G. Lizarralde , J. von Meding , and L. Bosher (2023) ‘Standardised indicators for “resilient cities”: the folly of devising a technical solution to a political problem’. International Journal of Disaster Resilience in the Built Environment. 14(4). pp. 514–535.

[disa12657-bib-0022] Chmutina, K. , W. Cheek , and J. von Meding (2021) ‘“Critique is not a verb”: is peer review stifling the dialogue in disaster scholarship?’. Disaster Prevention and Management. 31(4). pp. 387–397.

[disa12657-bib-0023] Chmutina, K. and J. von Meding (2022) ‘Towards a liberatory pedagogy of disaster risk reduction among built environment educators’. Disaster Prevention and Management. 31(5). pp. 521–535.

[disa12657-bib-0024] Chmutina, K. et al. (2024) ‘Journal of Disaster Studies: an inaugural discussion’. Journal of Disaster Studies. 1(1). Forthcoming.

[disa12657-bib-0025] Clark‐Ginsberg, A. (2021) ‘Risk technopolitics in Freetown slums: why community‐based disaster management is no silver bullet’. In J.A.C. Remes and A. Horowitz (eds.) Critical Disaster Studies. University of Pennsylvania Press, Philadelphia, PA. pp. 85–96.

[disa12657-bib-0026] Collini, S. (2012) What are Universities For? Penguin, London.

[disa12657-bib-0027] Collins, P.H. (2017) ‘On violence, intersectionality and transversal politics’. Ethnic and Racial Studies. 40(9). pp. 1460–1473.

[disa12657-bib-0028] Critchley, S. (2009) Ethics–Politics–Subjectivity: Essays On Derrida, Levinas & Contemporary French Thought. Verso, London.

[disa12657-bib-0029] Crowther, J. and M. Shaw (2017) ‘Solidarity in and against the academy’. In A. von Kotze and S. Walters (eds.) Forging Solidarity: Popular Education at Work. Sense Publishers, Rotterdam. pp. 203–211.

[disa12657-bib-0030] De Lissovoy, N. and A.L. Brown (2013) ‘Antiracist solidarity in critical education: contemporary problems and possibilities’. Urban Review. 45 (December). pp. 539–560.

[disa12657-bib-0031] de Sousa Santos, B. (2018) The End of Cognitive Empire: The Coming of Age of Epistemologies of the South. Duke University Press, Durham, NC.

[disa12657-bib-0032] Dean, A. , A. Venkataramani , and S. Kimmel (2020) ‘Mortality rates from COVID‐19 are lower in unionized nursing homes’. Health Affairs. 39(11). pp. 1993–2001. 32910688 10.1377/hlthaff.2020.01011

[disa12657-bib-0033] Dean, A. , J. McCallum , S. Kimmel , and A. Venkataramani (2021) ‘Iowa school districts were more likely to adopt COVID‐19 mask mandates where teachers were unionized’. Health Affairs. 40(8). pp. 1270–1276. 34339251 10.1377/hlthaff.2020.02518

[disa12657-bib-0034] Dean, A. , J. McCallum , S. Kimmel , and A. Venkataramani (2022) ‘Resident mortality and worker infection rates from COVID‐19 lower in union than nonunion US nursing homes, 2020–21’. Health Affairs. 41(5). pp. 751–759. 35442760 10.1377/hlthaff.2021.01687

[disa12657-bib-0035] Dean, J. (2019) Comrade: An Essay on Political Belonging. Verso, London.

[disa12657-bib-0036] Derrida, J. (2020) The Politics of Friendship. Verso, London.

[disa12657-bib-0037] Drescher, S. (2009) Abolition: A History of Slavery and Antislavery. Cambridge University Press, Cambridge.

[disa12657-bib-0038] Du Bois, W.E.B. (2007) In Battle for Peace: The Story of My 83rd Birthday. Volume 10. Oxford University Press, New York City, NY.

[disa12657-bib-0039] Durkheim, E. (1933) The Division of Labour in Society. First translation. Macmillan, New York City, NY.

[disa12657-bib-0040] English, B. (2015) ‘Providing insights into overcoming disaster’. The Boston Globe website. 31 August. https://www.bostonglobe.com/lifestyle/2015/08/31/resilience/u4TXnte3a9JKNs9D2K7AOO/story.html (last accessed on 19 August 2024).

[disa12657-bib-0041] Erikson, K. (1976) Everything in Its Path: Destruction of Community in the Buffalo Creek Flood. Simon and Schuster, New York City, NY.

[disa12657-bib-0042] Faas, A.J. (2022) In the Shadow of Tungurahua: Disaster Politics in Highland Ecuador. Rutgers University Press, New Brunswick, NJ.

[disa12657-bib-0043] Ferdinand, M. (2022) Decolonial Ecology: Thinking from the Caribbean World. Polity, Cambridge.

[disa12657-bib-0044] Foley, C. (2010) The Thin Blue Line. Verso, London.

[disa12657-bib-0045] Freire, P. (1998) Pedagogy of Hope: Reliving Pedagogy of the Oppressed. Continuum, New York City, NY.

[disa12657-bib-0046] Gagliano, J.C. (2021) ‘Solidarity for whom? Interrogating solidarity in geographic research methodologies’. *Geography* Compass. 15(8). Article number: e12587. 10.1111/gec3.12587.

[disa12657-bib-0047] Gaillard, J‐C. (2022) The Invention of Disaster: Power and Knowledge in Discourses on Hazard and Vulnerability. Routledge, London.

[disa12657-bib-0048] García Peña, L. (2022) Community as Rebellion: A Syllabus for Surviving Academia as a Woman of Color. Haymarket Books, Chicago, IL.

[disa12657-bib-0049] Gaztambide‐Fernández, R.A. (2012) ‘Decolonization and the pedagogy of solidarity’. *Decolonization: Indigeneity* , Education & Society. 1(1). pp. 41–67.

[disa12657-bib-0050] Genao, S. (2021) ‘Recasting solidarity: a call for radical engagement and academic unification’. International Journal of Qualitative Studies in Education. 36(8). pp. 1599–1611.

[disa12657-bib-0051] Gibson Graham, J. K. and G. Roelvink (2010) ‘An economic ethics for the Anthropocene’. Antipode. 41(S1). pp. 320–346.

[disa12657-bib-0052] Gill, R. (2018) ‘What would Les Back do? If generosity could save us’. International Journal of Politics, Culture, and Society. 31 (March). pp. 95–109.

[disa12657-bib-0053] Gilmore, R.W. (2022) Abolition Geography: Essays Towards Liberation. Verso, London.

[disa12657-bib-0054] Gramsci, A. (1971) Selections from the Prison Notebooks of Antonio Gramsci. International Publishers, New York City, NY.

[disa12657-bib-0055] Guidry, V.T. (2017) ‘In memoriam: Steve Wing’. Environmental Health Perspectives. 125(1). pp. A1–A2.

[disa12657-bib-0056] Hall, S. (1990) ‘Cultural identity and diaspora’. In P. Gilroy and R. Wilson Gilmore (eds.) Selected Writings on Race and Difference. Duke University Press, Durham, NC. pp. 222–237.

[disa12657-bib-0057] Harcourt, W. and I.L. Nelson (2015) ‘Introduction: are we green yet? And the violence of asking such a question’. In W. Harcourt and I.L. Nelson (eds.) Practising Feminist Political Ecologies: Moving Beyond the Green Economy. Zed Books, London. pp. 1–28.

[disa12657-bib-0058] Hechter, M. (2010) ‘Nationalism as group solidarity’. Ethics and Racial Studies. 10(4). pp. 415–426.

[disa12657-bib-0059] Herbert, W.A. , J. Apkarian , and J. van der Naald (2023) ‘Union organizing and strikes in higher education: the 2022–2023 upsurge in historical context’. In R. Milkman and J. van der Naald (eds.) The State of the Unions 2023: A Profile of Organized Labor in New York City, New York State, and the United States. City University of New York, New York City, NY. pp. 6–32.

[disa12657-bib-0060] Hill, D.D. (2019) ‘“Admirable or ridiculous?”: the burdens of Black women scholars and dialogue in the work of solidarity’. Journal of Feminist Studies in Religion. 35(2). pp. 5–21.

[disa12657-bib-0061] James, C.L.R. (2012) History of Pan‐African Revolt. PM Press, Binghampton, NY.

[disa12657-bib-0062] James, J. (2013) Seeking the Beloved Community: A Feminist Race Reader. State University of New York Press, Albany, NY.

[disa12657-bib-0063] Kaba, M. (2021) We Do This ’Til We Free Us: Abolitionist Organizing and Transforming Justice. Haymarket Books, Chicago, IL.

[disa12657-bib-0064] Kip, M. (2016) ‘Solidarity’. In K. Fritsch , C. O'Connor , and A.K. Thompson (eds.) Keywords for Radicals: The Contested Vocabulary of Late‐Capitalist Struggle. AK Press, Chico, CA. pp. 391–398.

[disa12657-bib-0065] Klinenberg, E. (2015) Heat Wave: A Social Autopsy of Disaster in Chicago. University of Chicago Press, Chicago, IL. 10.1056/NEJM200302133480721 12584383

[disa12657-bib-0066] Klutz, J. , J. Walker , and P. Walter (2020) ‘Unsettling allyship, unlearning and learning towards decolonising solidarity’. Studies in the Education of Adults. 52(1). pp. 49–66.

[disa12657-bib-0067] Koruth, M.A. (2023) ‘Rutgers deal could be “game‐changer” across U.S. as part‐time faculty win protections’. NorthJersey.com website. 15 April. https://www.northjersey.com/story/news/education/2023/04/14/unusual-rutgers-faculty-strike-has-academes-attention-across-us/70116703007/ (last accessed on 19 August 2024).

[disa12657-bib-0068] Kropotkin, P. (1902) Mutual Aid: A Factor of Evolution. McClure Phillips & Co., New York City, NY.

[disa12657-bib-0069] Lorde, A. (2012) Sister Outsider: Essays and Speeches. Clarkson Potter and Ten Speed Press, New York City, NY.

[disa12657-bib-0070] Loveday, V. (2018) ‘The neurotic academic: anxiety, casualisation, and governance in the neoliberalising university’. Journal of Cultural Economy. 11(2). pp. 154–166.

[disa12657-bib-0071] Lynd, S. (1984) ‘Communal rights’. Texas Law Review. 62(8). pp. 1417–1441.

[disa12657-bib-0072] Lynn, D. (2017) ‘The Marxist proposition, Claudia Jones, and Black nationalism’. African American Intellectual History Society website. 1 November. https://www.aaihs.org/the-marxist-proposition-claudia-jones-and-black-nationalism/ (last accessed on 19 August 2024).

[disa12657-bib-0073] Mann, G. and J. Wainwright (2020) Climate Leviathan: A Political Theory of Our Planetary Future. Verso, London.

[disa12657-bib-0074] Marshall, B.L. and A. Witz (2004) ‘Introduction: feminist encounters with sociological theory’. In B.L. Marshall and A. Witz (eds.) Engendering the Social: Feminist Encounters with Sociological Theory. Open University Press, Maidenhead. pp. 1–16.

[disa12657-bib-0075] Mbembe, A. (2021) Out of the Dark Night: Essays on Decolonization. Columbia University Press, New York City, NY.

[disa12657-bib-0076] McAlevey, J.F. (2016) No Shortcuts: Organizing for Power. Oxford University Press, New York City, NY.

[disa12657-bib-0077] McKittrick, K. (2021) Dear Science and Other Stories. Duke University Press, Durham, NC.

[disa12657-bib-0078] Miles, S. , A. Renedo , and C. Marston (2022) ‘Reimagining authorship guidelines to promote equity in co‐produced academic collaborations’. Global Public Health. 17(10). pp. 2547–2559. 34520317 10.1080/17441692.2021.1971277

[disa12657-bib-0079] Mohanty, C.T. (2003) ‘“Under Western Eyes” revisited: feminist solidarity through anticapitalist struggles’. Signs. 28(2). pp. 499–535.

[disa12657-bib-0080] Morgan, G. and V. Pulignano (2020) ‘Solidarity at work: concepts, levels and challenges’. Work, Employment and Society. 34(1). pp. 18–34.

[disa12657-bib-0081] Morimoto, R. (2023) Nuclear Ghost: Atomic Livelihoods in Fukushima's Gray Zone. University of California Press, Berkeley, CA.

[disa12657-bib-0082] Moroz, J. and O. Swabovski (2017) ‘Solidarity, dark solidarity, the commons and the university’. Power and Education. 9(2). pp. 145–158.

[disa12657-bib-0083] Nancy, J‐L. (2016) The Disavowed Community. Fordham University Press, New York City, NY.

[disa12657-bib-0084] Nelson, C. (1996) A Yale Strike Dossier: *Vol* 14, No. 4. *Social Text*. 49. Duke University Press, Durham, NC.

[disa12657-bib-0085] Nightingale, J. (2015) The Concept of Solidarity in Anarchist Thought. A doctoral thesis submitted in partial fulfilment of the requirements for the award of Doctor of Philosophy at Loughborough University. https://repository.lboro.ac.uk/articles/thesis/The_concept_of_solidarity_in_anarchist_thought/9466781 (last accessed on 19 August 2024).

[disa12657-bib-0086] Öniş, Z. (1991) ‘The logic of the developmental state’. Comparative Politics. 24(1). pp. 109–126.

[disa12657-bib-0087] Power, M. and J.A. Charlip (2009) ‘Introduction: on solidarity’. Latin American Perspectives. 169(36). pp. 3–9.

[disa12657-bib-0088] Purdum, C. et al. (2021) ‘No justice, no resilience: prison abolition as disaster mitigation in an era of climate change’. Environmental Justice. 14(6). pp. 418–425.

[disa12657-bib-0089] Reay, D. , G. Crozier , and J. Clayton (2009) ‘“Strangers in paradise”? Working‐class students in elite universities’. Sociology. 43(6). pp. 1103–1121.

[disa12657-bib-0090] Remes, J.A.C. (2012) ‘Solidarity, citizenship, and the opportunities of disasters’. In D. Katz and R.A. Greenwald (eds.) Labor Rising: The Past and Future of Working People in America. New Press, New York City, NY. pp. 143–153.

[disa12657-bib-0091] Remes, J.A.C. (2016a) ‘Books in the basement: formal movement learning and turning discontent into understanding (a response to Aziz Chourdry)’. Explorations in Adult Education: An Occasional Papers Series. 4. pp. 61–68.

[disa12657-bib-0092] Remes, J.A.C. (2016b) Disaster Citizenship: Survivors, Solidarity and Power in the Progressive Era. University of Illinois Press, Urbana, IL.

[disa12657-bib-0093] Remes, J.A.C. and A. Horowitz (2021) Critical Disaster Studies. University of Pennsylvania Press, Philadelphia, PA.

[disa12657-bib-0094] Richards, L. (2010) Union‐Free America: Workers and Antiunion Culture. University of Illinois Press, Urbana, IL.

[disa12657-bib-0095] Rorty, R. (1989) Contingency, Irony and Solidarity. Cambridge University Press, Cambridge.

[disa12657-bib-0096] Santos, A.C. (2012) ‘Disclosed and willing: towards a queer public sociology’. Social Movement Studies. 11(2). pp. 241–254.

[disa12657-bib-0097] Scholz, S.J. (2008) Political Solidarity. Pennsylvania State University Press, University Park, PA.

[disa12657-bib-0098] Servet, J‐M. and B. Tinel (2020) ‘The behavioral and neoliberal foundations of randomizations’. Strategic Change. 29(3). pp. 293–299.

[disa12657-bib-0099] Seymour, R. (2012) The Liberal Defence of Murder. Verso, London.

[disa12657-bib-0100] Sheppard, E. (2021) ‘“I'm not saying this to be petty”: reflections on making disability visible while teaching’. In N. Brown (ed.) Lived Experiences of Ableism in Academia: Strategies for Inclusion in Higher Education. Policy Press, Bristol. pp. 185–196.

[disa12657-bib-0101] Shukla, P.R. et al. (2022) ‘Summary for policymakers’. In P.R. Shukla et al. (eds) *Climate Change 2022: Mitigation of Climate Change*. Working Group III contribution to the Sixth Assessment Report of the Intergovernmental Panel on Climate Change. Cambridge University Press, Cambridge. pp. 1–48.

[disa12657-bib-0102] Simone, A.M. (2004) ‘People as infrastructure: intersecting fragments in Johannesburg’. Public Culture. 16(3). pp. 407–429.

[disa12657-bib-0103] Southam, T. (2021) ‘Academics as allies and accomplices: practices for decolonized solidarity’. Anthropology and Aging. 42(2). pp. 150–165.

[disa12657-bib-0104] Spade, D. (2020) Mutual Aid: Building Solidarity During this Crisis (and the Next). Verso, London.

[disa12657-bib-0105] Stein, H. , S. Cunningham , and P. Carmody (2021) ‘The rise of “behavioral man”: randomized controlled trials and the “new” development agenda’. Human Geography. 14(1). pp. 62–75.

[disa12657-bib-0106] Stjernø, S. (2005) Solidarity in Europe: The History of an Idea. Cambridge University Press, Cambridge.

[disa12657-bib-0107] Taylor, A. (2021) Remaking the World: Essays, Reflections, Rebellions. Haymarket Books, Chicago, IL.

[disa12657-bib-0108] The Care Collective (2020) The Care Manifesto: The Politics of Interdependence. Verso, London.

[disa12657-bib-0109] Tuck, E. and K.W. Yang (2012) ‘Decolonization is not a metaphor’. Decolonization: Indigeneity, Education & Society. 1(1). pp. 1–40.

[disa12657-bib-0110] Tuck, E. and K.W. Yang (2014) ‘R‐words: refusing research’. In D. Paris and M.T. Winn (eds.) Humanizing Research: Decolonizing Qualitative Inquiry with Youth and Communities. Sage Publications, Thousand Oaks, CA. pp. 223–247.

[disa12657-bib-0111] UNISDR (United Nations Office for Disaster Risk Reduction) (2015) Sendai Framework for Disaster Risk Reduction 2015–2030. UNISDR, Geneva.

[disa12657-bib-0112] Vachon, T.E. , M. Wallace , and A. Li (2023) ‘Unions, democracy, and Trump: deconstructing the COVID‐19 vaccination crisis of 2021’. Social Science Research. 115 (September). Article number: 102918. 10.1016/j.ssresearch.2023.102918. 37858361

[disa12657-bib-0113] von Meding, J. and K. Chmutina (2023) ‘From labelling weakness to liberatory praxis: a new theory of vulnerability for disaster studies’. Disaster Prevention and Management. 32(2). pp. 364–378.

[disa12657-bib-0114] Wark, M. (2021) Capital is Dead: Is This Something Worse? Verso, London.

[disa12657-bib-0115] Watanabe, C. (2021) ‘Translating disaster knowledge from Japan to Chile: a proposal for incompleteness’. In J.A.C. Remes and A. Horowitz (eds.) Critical Disaster Studies. University of Pennsylvania Press, Philadelphia, PA. pp. 165–183.

[disa12657-bib-0116] Wilder, C.S. (1998) ‘The rise and influence of the New York African Society for Mutual Relief, 1808–1865’. Afro – Americans in New York Life and History. 22(2). pp. 1–7.

[disa12657-bib-0117] Williams, D.A. (2023) ‘Let's build our own house: political art and the making of Black and Muslim worlds’. Southern Cultures. 29(2). pp. 48–67.

[disa12657-bib-0118] Wisner, B. (2019) ‘Disaster studies at 50: time to wear bifocals?’. In J. Kendra , S.G. Knowles , and T. Wachtendorf (eds.) Disaster Research and the Second Environmental Crisis. Springer Nature Switzerland AG, Cham. pp. 47–68.

[disa12657-bib-0119] Wisner, B. (2025) ‘Reflections on disaster risk and suffering’. In K. Chmutina , R. Fuentealba , and D. Rivera (eds.) Principles and Concepts of Disaster Risks. World Scientific Publishing Co., Singapore. Forthcoming.

[disa12657-bib-0120] Wright, E.O. (2021) How to Be an Anti‐Capitalist in the Twenty‐First Century. Verso, London.

